# Self-injurious behavior: A clinical appraisal

**DOI:** 10.4103/0019-5545.44754

**Published:** 2008

**Authors:** K. Nagaraja Rao, C. Y. Sudarshan, Shamshad Begum

**Affiliations:** Department of Psychiatry, JJM Medical College, Davangere, Karnataka - 577 004, India

## Abstract

A case series of self-injurious behavior (SIB) encountered in a General Hospital setting has been described. Limitations of current definition of SIB are explained. SIB is not a single clinical entity and it occurs in various psychiatric syndromes with wide range of psychopathology. Based on clinical criteria, a classification of SIB into three groups has been proposed viz 1) Mild and isolated form, 2) Moderately severe and repetitive form, and 3) Very severe and isolated form. Psychodynamic, cognitive and neurochemical explanations of SIB have been reviewed. Frustration, aggression and impulsivity appearing in helpless situation appear to be a common script across most of these models of explanations. Severity of injury seems to be determined by severity of psychopathology. Site of injury appear to have symbolic significance for a particular patient. Understanding some of these clinicopsychopathological issues helps in management of these cases.

## INTRODUCTION

Cases of self-injurious behavior (SIB) are common in routine clinical practice. It is defined as commission of deliberate injury to one's own body done without the aid of the other person and is severe enough to cause tissue damage/scarring. Acts that are associated with sexual arousal and acts committed with conscious suicidal intent are excluded.[1] It has been variously termed as deliberate self-harm (DSH), self-harm, superficial-moderate self-mutilation, parasuicide, self-wounding,[2] auto-aggression and purposive accidents.[3] Though various authors have given working definitions for their terms sometimes these terms are used loosely. Pattison and Kahan[4] have proposed the term DSH syndrome for cases of SIB having low lethality (non-suicidal) usually beginning in adolescence persisting for decades resulting in personal and social morbidity. Feldman[3] distinguishes self-mutilation from SIB; according to him, self-mutilation is intentionally damaging a part of own body apparently without a conscious intent to die, and SIB is an array of behaviors ranging from self-biting and hitting to pica occurring in psychiatric conditions. Various forms of self-injury have been described across a spectrum of psychiatric disorders. At times, persons with no obvious psychiatric disorders injure themselves. Some types of injuries are culturally sanctioned. Nature of injury varies from mild to very severe form. It could be isolated or repetitive. Mild and moderately severe form of injuries include banging the head, kicking the limbs, skin picking, carving words on the skin, sand papering the face, dripping acid on the hands, biting, burning, cutting, pulling out finger and toe nails, chewing fingers and so on. Very severe forms include mutilation of various body parts such as removal of eyes, ears, genitalia, tongue, teeth, digits and limbs. At times, severe mutilations may be associated with auto cannibalism and auto surgery.[5]

In this paper, a case series of deliberate self-injury encountered in an Indian general hospital setting is reported along with discussion of certain clinical, sociocultural and psychopathological issues.

## CASE HISTORIES

### A) Mild and Isolated form of SIB:

1. An 18-year-old male student had inscribed his girl friend's name “Neethu” and the number “143,” which symbolically meant “I love you,” (representing number of letters in each word) on his chest to prove his love towards her. He was dejected because of rejection by the girl liked by him. He had no diagnosable psychiatric disorder.
2. A 19-year-old female medical student branded her abdomen with a burning cigarette snatched from her lover to prove her love towards him, which was questioned by him during a quarrel. She had no diagnosable psychiatric disorder
3. A 20-year-old female having been dejected in love affair had slashed her wrists with a scalpel.
4. A 20-year-old male who was in love with a girl had cut a small part of his left thumb to prove his unstinting love towards her. He had no diagnosable psychiatric illness at that time, although he developed mild depression after few years.
5. A 25-year-old male sought consultation for mild depression. Past history revealed that 2 years back, he had felt sad when his younger sister got married. He had written her name on his left forearm with burning match sticks, which had scarred his forearm. Though he was happy that his sister was getting married, he was sad that she was going away from him. He said he was deeply attached to her as she had looked after the whole family after death of their mother many years ago. However, he said that his sadness at that time did not need treatment.
6. A 23-year-old male had tattooed initials of a girl on his left fore arm with whom he was in love. After few years, one day when the girl did not respond to him properly, he placed camphor on the tattoo mark and burnt the camphor in a bid to erase the tattoo mark. He was a social drinker to begin with and later developed alcohol dependence with depressive features.
7. A 25-year-old male who had parasomnia was found to have tattoo mark on his left hand, which was scarred due to burning. On enquiry, he revealed that he had tattooed initials of the girl whom he in love with. One day when the girl spurned his advances towards her, he attempted to erase the initials by burning camphor on it after consuming alcohol. He had no diagnosable psychiatric disorder
8. A 25-year-old male had burnt his left upper limb with cigarette butts in a span of 3 days. The limb had appearance of a deer skin. The reason for the act was that his parents had not approved his particular business plan. He had no diagnosable psychiatric disorder
9. An 18-year-old female nursing student had burnt her right palm with a burning candle after she was admonished for lapse in her duty by her superiors. She had no diagnosable psychiatric disorder.
10. A 32-year-old teacher had burnt camphor on his right palm resulting in a scar as his wife had denied sex when he wanted. His wife was found to have lack of sex desire.

### B) Moderate and repetitive form of SIB:

1. A 20-year-old girl had scars on her forearms. She had inflicted them whenever she felt frustrated, anxious or when her demands were not met. On evaluation, she was found to have borderline personality disorder.
2. A 23-year-old married female used to get angry for trivial matters and throw temper tantrums frequently. She was over concerned with cleanliness, orderliness and was highly demanding. She wished to have her say always. She had not liked job of her husband as Television mechanic as she thought that it was a menial job. Frequent quarrels would ensue over this issue. During quarrels and whenever her demands were not met, she used to brand herself with hot spoon over her fore arms and face. She was diagnosed to have borderline personality disorder.
3. A 22-year-old girl inflicted injury on her left forearm by sharp blade on different occasions whenever her boyfriend did not meet her demands. On evaluation, she was found to have borderline personality disorder with depressive features.
4. An 18-year-old girl was admitted with complaints of decreased interest in studies, irritability, aches and pains, episodes of loss of awareness. She was diagnosed to have major depressive disorder with histrionic personality. Her complaints followed increase in work load, not being granted permission to go to college, while her sister was permitted to attend college. She felt that she was given less attention as compared to her sister and was unloved. This led to frequent quarrels and during these quarrels, she used to inflict injuries on her forearms with sharp blade.
5. A 20-year-old female developed features of major depressive disorder following failure in love affair. She had feelings of guilt, hopelessness, worthlessness, lack of self-esteem, depressed mood and ideas of suicide. She used to slash her left forearm whenever she had difficulties with her boy friend. During present admission, she had attempted suicide by consuming 100 ml of phenol and simultaneously injecting 3-4 ml of it intravenously. She also had slashed her left wrist with a scalpel.
6. A 20-year-old male presented with large and wide scars of deep incised wounds on his left forearm inflicted using a sharp blade whenever he felt intense anger. He said he had difficulty in controlling his anger in any other way.
7. A 24-years-old male diagnosed to have alcohol dependence had inflicted several cuts on his forearms with a blade on two occasions under the influence of alcohol. On both occasions, he was humiliated by restricting entry into one of his relative's and his own house while he was under the influence of alcohol. When he was sober he said that he had inflicted injury due to his helplessness and vowed that he would take revenge by inflicting similar and double the number of injuries on his relative irrespective of consequences involved.
8. A 25-year-old male was admitted for alcohol de-addiction. On examination he was found to have multiple scars on ventral aspects of his both forearms. On enquiry, it was revealed that he had inscribed his fiancé's name on his left fore arm. The girl was married off to another person. Then, he had tried to erase her name from his forearm using a blade and in addition inflicted several cuts on his right forearm. Both these acts were done under the influence of alcohol.
9. A 26-year-old male was admitted with history of alcohol dependence. He had history of repeatedly injuring himself by cutting his forearm with a sharp blade whenever his demands were not met. He had comorbid depression on examination.
10. A 9-year-old girl was brought with complaints of rubbing her genital by her hands and against the bed. She also had history of repetitive scratching and skin picking. She was dull in studies. She was found to have moderate mental retardation.

### C) Very severe and Isolated form of SIB:

1. A 20-year-old male who had schizophreniform psychosis of 3-month duration had bitten his right index finger which resulted in bleeding and a wound needing stitches during acute phase of his illness. He had no delusions or hallucinations on evaluation.
2. A 26-year-old married male patient had an altercation with a religious head accusing him of performing black magic on him. He was diagnosed to have paranoid schizophrenia. Patient reported that after black magic he got mad, was not under his own control and felt like taking away his life. With the suicidal intention, he cut his scrotum on the right side resulting in a wound four inches long, which needed surgery.
3. A 20-year-old boy was seen with features of disorganized schizophrenia in the form of gross disorganized behavior, poor personal hygiene, silly giggling, poverty of speech, poor motivation and flat affect of 3-year duration. During a follow up, the patient's mother reported that patient deeply incised his left cheek and both upper eyelids with a blade. There were linear scar marks on the cheek and eyelids. Patient gave no explanation for it.
4. A 20-year-old male following the death of his father became dull, disinterested, sleepless, and pulled out his right eye ball, after an argument with his brother over property issues. On examination, patient was retarded and depressed. There were no delusions and hallucinations. At that time, he was diagnosed as a case of major depressive disorder. Subsequently, he developed an episode of mania.
5. A 45-year-old male who had past history of an episode of depression had presented with complaints of feeling sad, anxious and ideas of suicide. He was found to have delusion of guilt and persecution. He had cut his penis with intent to die and to get over his guilt.
6. An 82-year-old male had history of depressive features for 9 months overtly due to inability to live with his son during the last phase of his life. His son had moved to a new place to take up a new job. He had amputated his penis and testes using a razor blade with the intention of suicide and succumbed to it.
7. An 18-year-old male presented with complaints of sadness of one year duration because of having to perform an inferior job compared to one that of his brother. He amputated his testes and penis with suicidal intention. He was found to have mild mental retardation. His IQ was 52. He had no other psychiatric illness.
8. A 30-year-old male with alcohol dependence of 10-year duration stopped drinking suddenly and 3 days later he was fearful, shabbily dressed, tremulous, started talking as if he was conversing with some one and told that some people were chasing him. That night he slept poorly and next day started telling that something is stuck in his lower left incisor tooth and pulled out the tooth with his hand. Patient claimed amnesia for this event after he recovered. He was diagnosed to have delirium tremens.
9. A 50-year-old male patient had alcohol dependence. Four days after his last drink, he was brought to hospital with history of sleeplessness, fearfulness, hearing voices, and telling that others are coming to kill him. Refusing admission, patient went back home and cut his tongue horizontally. He claimed no memory for his act. He was diagnosed to have alcohol induced psychosis.
10. A 20-year-old lady delivered in a hospital by caesarian section. On the fourth postpartum day, she developed fever and started behaving abnormally. She was restless, sleepless, suspicious, talked irrelevantly and was disoriented. Next night she pulled out four of her sutures and was found bleeding. It needed resuturing. Later patient claimed amnesia for her act. She was diagnosed to have acute organic psychosis.
11. A 35-year-old known epileptic was seen in emergency ward with self-inflicted multiple deep incised wounds on his abdomen. On recovery, he said that he did not know how he had done it and why he had done it. It was an act of automatism fallowing an attack of epilepsy.
12. A 29-year-old Hindu male reported himself to the emergency ward after having chopped off his penis with a chisel at its base. On examination he was found to have erectile disorder with depressive features. He had mutilated his penis to avoid marriage fearing humiliation of sexual nonperformance.

## DISCUSSION

### CLINICAL ISSUES

#### 1) Definition

There are several definitions for SIB. The definition by Winchel and Stanley[1] is well accepted.[2,3] The definition excluding cases of conscious suicidal intention seems to be too restrictive. There are many cases of SIB reported in literature with suicidal intention, especially in severe form.[6–8] In the present series, in case numbers 12 to 14, suicidal intention is evident. Further, it is estimated that around 25% of suicides are preceded by nonfatal self-harm.[9] It is also claimed that an episode of self-harm with recent discharge from in-patient psychiatric care is a major risk factor for suicide.[10] Therefore, it is prudent to restrict the definition of SIB to phenomenological explanation of “what the person did?” and should not include the intentions for the act “why the person did?,” although latter considerations are of utmost importance for understanding psychopathology and planning treatment. SIB has also been called deliberate self-harm (DSH). There have been arguments to drop the term deliberate and retain the term self-harm because of the heterogeneous nature of this phenomenon.[11,12] There have been suggestions to bring self-poisoning and self-injury under the rubric of self-harm.[13] This suggestion broadens the definition extremely and excludes the significance of self-injury by clubbing it with self-poisoning, which has no component of overt physical injury. Therefore, we propose the following definition “SIB is commission of deliberate injury to one's own body without the aid of the other person causing tissue damage.”

#### 2) Clinical classification

SIB is a heterogeneous group of behavior because of which it is classified in several ways. There have been classification based on (a) Severity:[3] major and minor, mild and severe, (b) Frequency (number per year): 1-3 times - mild, 4-11 times - moderate, more than 11 times - severe.[14] (c) Site: Self-cutting of skin with subtypes low-lethality delicate cutters and high-lethality coarse cutters, occular self-mutilation and genital self-mutilation,[3] (d) Psychiatric diagnoses; with mental retardation, with psychosis, with personality disorders.[3]

SIB has also been considered as a behavioral expression of specific genetic disorders and as state-related behavior that accompanies certain psychiatric disorders.

Combining severity, repetitiveness and diagnosis, cases described in this paper could be classified broadly into three clinical categories. The first group consists of mild and isolated forms. Majority of such cases occurred in people with no overt psychiatric diagnosis or with subthreshold psychiatric disorder. The second group consists of moderately severe and repetitive form of SIB. Most of such cases were seen in non-psychotic conditions. The third group consists of very severe and isolated form of SIB, usually amounting to mutilation. Such cases were invariably found in psychotic patients and were associated with severe psychopathology such as delusions, hallucinations, suicidal intentions, and confusion.

We are aware that it is difficult to compartmentalize a clinical symptom occurring in conditions ranging from normalcy to severe psychiatric disorders. However, majority of cases could be categorized into one of the three groups. Hence, this classification has some clinical relevance. There is also some support for this classification in the literature, which will be discussed under psychiatric diagnoses.

#### 3) Psychiatric diagnoses

In the present case series, case numbers 1 to 10 had mild and isolated or less frequent self-injuries. Day-to-day activities of these persons were not hampered. There was no diagnosable psychiatric illness in them at the time of act. The occurrence of SIB in normal people is supported by the findings that Self-injurious behavior has been reported in 4% of non-clinic general population[15] and 14% of college students[16] besides 35% of college students having reported at least one self-harm behavior in their life time.[17] Case numbers 4, 5 and 6, though had no diagnosable psychiatric illness at the time of SIB, developed depression and alcohol dependence after few years. This may mean that either they had predisposition to or sub-threshold psychiatric illness.

From our pool of cases, case numbers 11, 12, 13 (borderline personality disorder); 14 and 15 (major depressive disorder); 16 (impulse control disorder); 17, 18, and 19 (alcohol dependence); and 20 (mental retardation) had repetitive and moderately severe self-injuries. Case numbers 13 and 19 had comorbid depression. Case number 14 had comorbid histrionic personality.

In the literature, moderately severe and repetitive SIB has been reported in most of the personality disorders except in obsessive compulsive personality disorder.[2].It has been reported most frequently in borderline personality disorder (BPD)[18] so much so that SIB is a diagnostic criterion for BPD (DSM). It has also been reported in impulse control disorder, Mental retardation[19–21] infantile autism,[22,23] factitious disorder,[3,24] prison inmates,[25] substance abuse and depressive disorder.[26,27] Cases belonging to this group usually are brought for psychiatric help and need active and long-term psychiatric management. SIB in cases of substance abuse,[28] and factitious disorder[29] have been described in Indian setting. The earlier notion that sexual abuse in children results in self-injurious behavior later is not supported by empirical evidence. It is probable that both are related as they are correlated with same psychiatric risk factors.[30]

Head banging during arguments and quarrels when one is overwhelmed with frustration and helplessness may also belong to second category. Many cases of neuroses and alcohol dependence bang their head and beat their chest when frustrated. In fact there is a proverb which means “the weak and helpless injure themselves.” However, such cases cannot be included under SIB, unless there is tissue damage.

Case numbers 21 (schizophreniform disorder); 22 (paranoid schizophrenia); 23 (disorganized schizophrenia); 24 (bipolar disorder); 25 and 26 (major depressive disorder); 27 (mental retardation); 28 and 29 (alcohol dependence); 30 (OBS); 31 (epilepsy); and 32 (erectile disorder) had isolated and very severe form of SIB.[31–34]

The two most common forms of severe self-injuries are genital mutilation and self-enucleation. Other forms of severe injuries are dental extraction, cutting or biting of tongue, cutting of breast, deep abdominal wound and amputation of other body parts. Injuries secondary to self-induced abortions and self-insertion of foreign bodies by children also lead to various forms of genital injuries. Though severe forms of self-injuries are isolated events, repetitive mutilations have been described on rare occasions.[6,35]

Genital self-mutilation was first reported by Storch in 1901.[36] Since then a number of cases of mutilation have been reported. Very severe and isolated form of SIB is usually described in psychotic disorders.[37,7,38] Schizophrenia secondary to delusions and hallucinations,[39,40] depression,[41] and mental retardation.[42,43]

There have been reports of self-mutilations in non-psychotic cases too.[44–46] Case no 32 is a severe and isolated form of self-injury in a nonpsychotic state.[33] Cases of severe mutilation in response to religious delusions[47] and as a means to relieve symptoms of urinary discomfort[48] and a case of self-induced bleeding to relieve pain in a case of delusional disorder[49] have also been reported in Indian setting. Authors are aware of 2 cases of self-mutilation published in national newspapers. In the first case, a middle-aged male diabetic who had developed gangrene in one of his foot was advised amputation. He attempted to amputate his foot by putting it across an electric train as an effort at self-surgery reportedly to save on hospital expenses and died in that process. In another case, a middle-aged male had cut his tongue when his leader did not get elected in an election as he did not want to speak about it. In these 2 cases, there was no mention of any other abnormal behaviors. However, as these cases were not evaluated clinically, normalcy is more a conjecture than a firm diagnosis.

Although severe mutilations occur as an isolated event, there have been instances of previous attempts at self-mutilation.[37]

SIB have also been described in many medical conditions such as epilepsy[50] encephalitis,[51] diabetes,[52] hypothyroidism[35] and eating disorders[53,26,27] and prison population.[54,55] Case number 31 described in the present series had SIB during an attack of epilepsy.

We did not come across any cases of eating disorders with SIB, which is reported to be of frequent occurrence in western population.[53,27] This is probably due to low prevalence of such disorder in Indian culture. We also did not come across cases of SIB in prison population. Prison authorities probably might be considering the matter as trivial and that manipulative behavior needs firm action.

#### 4) Clinical variables

In this study, mild and moderately severe form of SIB occurred in younger patients (mean age 21 years, range 9-25 years) than in severe form (mean age 33.6 years, range 18-82 years). Almost one-third of patients aged between 8 years to 18 years having first episode psychosis had recent history of self-harm just prior to admission.[56] In our series, the first and second groups had more number of females, whereas the third group had more number of males. The first and second groups had injured the skin more often, whereas the third group had injured their genitals more often. The relationship between repeated self-harm and gender is debated. Young et al.[57] reported that repeated self-harm was unrelated to gender, while Rajwal and Gash[58] discounted the old myth of higher proportion of females repeatedly harming themselves.

#### 5) Sociocultural issues

Western literature has drawn attention to undertones of mythological and religious explanations for SIB.[59–61] Even in Indian mythology, there have been allusions to SIB in various forms, including self-enucleation (Bedara Kannappa), amputation of thumb (Ekalavya) and amputation of breast (Kannagi). Practices such as piercing the tongue with a sharp object, walking on fire and thorns, burning camphor on open palm, tonsuring the head, rolling in prostrated body position as methods for propitiation of God are being practiced sporadically even today. Indian History is replete with instances where in a king going on a war expedition being applied blood mark on the fore head (rakta tilika) by his queen from the blood drawn from her thumb. Putting a signature or writing a message using one's own blood is considered as a method of proving loyalty or determination. There are sufficient examples of self-immolation to prove love towards one's beloved and loyalty towards one's hero. In contemporary time, tattooing name of a person on one's body is considered as a sign of love. Tattooing of the penis is seen in western cultures among drug addicts and prison inmates.[3] Implanting plastic spherules under the skin of the penis in Japanese racketeers to enhance sexual excitement is in practice.

In many societies, there have been culturally sanctioned injuries. Nose and ear piercing, male and female circumcision are examples of injurious behavior approved by self on religious or cultural grounds. These types of ceremonial mutilations though often not actually self-inflicted are submitted to cooperatively if not eagerly[62] sometimes with ignorance and helplessness. At times, there have been efforts at reconstruction of mutilated parts. Thus, cultural factors do influence SIB at least partly.

## PSYCHOPATHOLOGY

Various psychopathological explanations have been offered to describe SIB.

### Psychoanalytical explanation

The psychoanalytical explanations offered for cases of the first group include forming an identity, responding to self-hatred or guilt and discharging sexual feeling and relief from tension.[63] On some occasions, it could be an adaptive behavior stemming from archaic biological affect in desperate situation.[3]

Psychoanalytical explanations offered for cases of the second group are use of primitive defense mechanisms such as denial, splitting and projective identification linked to preoedipal developmental pathology,[64] sadistic sexual abuse by an older person early in life followed by extreme guilt in later life, with self-injury aiding in relief of tension.[65] Further, in depressive disorder, SIB has been linked to castration and explained as a process of failure to resolve oedipal conflict, repressed impulses, self-punishment[61] and focal suicide.[66] Thus, self-injury may offer direct sadistic and masochistic gratification or an atonement for the gratification, symbolic castration or penetration, masturbation or a masturbation equivalent with flowing blood giving orgasmic relief of tension.[67] Flowing blood may serve many functions such as offering a symbolic solution of menstrual conflict, warm blood giving a feeling of security blanket, and letting the blood out equated with archaic blood (poison) letting practice.

Aggression turned inwards is one of the major psychodynamic theories in depression.[31] The self may be the target of aggression as certain aspects of self such as inescapable destructive impulsive or compulsive traits become the object of hatred by the rest of the personality. Leibowitz[68] emphasized the interpersonal losses that often appear to precede bouts of SIB in some cases. He and Klein called this phenomenon as rejection sensitivity occurring in hysteroid dysphoric form of atypical depression. In some cases of SIB, internalization may not fully neutralize the aggression. Such situation may also arise in some cases of attempted suicide, which has been referred to as Menninger's triad consisting of Individual killing himself, fulfilling wish to die and symbolically murdering somebody.[62]

In cases akin to the third group, aggression turned inwards is a frequent explanation, especially in depression. Other psychoanalytical explanations are failure to resolve Oedipus complex, Oedipus self-blinding equated with self-castration, castration fears, repressed impulses and self-punishment.[66] Further, self-mutilation has been linked to focal suicide where in the mutilated part is considered to represent the condensation of the self, where it allows one to kill the devilish responsible self and still live.[69,70]

By using interpersonal dimension, Podvoll et al. suggested that patient protects himself from omnivorous and engulfing demands on others by a flight from deeply dependent, symbolic wish towards a primitive love object to a reliance on an autoerotic use of one's own body. He further emphasized that by redirecting aggression toward oneself, the rage and explosiveness finds a secure home on a fixed and seemingly indestructible object.[71]

### Descriptive psychopathology

In the first group of cases, the intent usually is to appease, gain approval or acceptance from others probably emerging out of strong dependency needs.

In cases belonging to the second group, mounting tension with depersonalization, anxiety and depression, impulsivity and aggression have been correlated to self-injury.[72] While describing the role of mounting tension Leibenluft et al. has described five stages of self-injuring act 1) The precipitating event (e.g. Loss of significant relationship), 2) Escalation of dysphoria, 3) Attempts to forestall the self-injury, 4) Self-injury, and 5) Aftermath (e.g., relief from tension).[65] However, the relief may be followed by self-hatred, disappointment, fear of consequences or a sense of badness resulting in patient's refusal to recall conflicts that precipitated the cutting[73–75] Pain is often absent during the cutting, probably due to transformation of psychological torment into tolerable physical sensation or the act confirming presence of feelings through pain.[76] Flowing of one's own blood is often sought with no interest in stale blood, menstrual blood or in others blood.[77]

While considering the affective state though both anxiety and depression have been found in cases of SIB, it is argued that anxiety is a predominant affect to be targeted for treatment. This view is based on the finding that patients of SIB despite experiencing negative effects have capacity to experience positive affect. And also according to the tripartite model, both anxiety and depression are associated with negative effects but depression alone is associated with decrease in positive affect. It is also suggested that anxiety and depression in SIB is accompanied with more of somatization and cognitive disturbance rather than vegetative disturbances.[72] It is further proposed that mounting anxiety may be either a direct precipitant of SIB or the final pathway to a variety of thoughts, affects and experiences that trigger SIB.

Two more important factors in SIB are impulsivity and aggression. It has been found that despite scoring high on impulsivity, it is the greater aggression combined with poor impulse control that causes SIB rather than impulsivity alone. Impulsivity has been found to be more prominent in less aggressive impulsive behavior such as promiscuity, gambling, overspending, overeating, oversleeping etc. Based on this line of thinking it is opined that if Impulse Control Disorder is subdivided into aggressive and non- aggressive type SIB falls into aggressive type along with intermittent explosive disorder, pyromania, and trichotillomania and pathological gambling, kleptomania fall under non-aggressive forms.[72] Currently cases of self-injurious behavior that do not meet the criteria for any specific impulse control disorder or for another mental disorder have been classified as impulse control disorder NOS in DSM-IV. Epidemics of self-injury reported in correctional institution for teenage offenders are believed to indicate group identification. Impulsivity inherent in other forms of self-injurious behavior may be absent in such cases.[78,3] In fictitious disorder, this behavior is employed to escape from unpleasant situations and duties and to gain special protective custody in hospitals and prison.[79,80]

In addition to above psychopathological mechanisms in cases of the third group, additional psychopathological mechanisms operating include command hallucinations, religious (Klingsor syndrome), hypochondriac, bizarre or dysmorphophobic delusions (Von-Gogh syndrome), delusional parasitosis, autistic thinking, sexual transgression, eroticism, punishment for guilt, suicidal ideation, internalized aggression and cognitive deficits.

Nonpsychotic genital self-mutilation has been reported in character disorders as a rageful feeling towards oneself or others. Transsexual males sometimes premeditate their own gender conversion surgery, especially when they can not afford cost of surgery.[37] In such cases, we have proposed that severe form of mutilation is a form of psychotic solution without recourse to much simpler nonpsychotic solution available for a rational mind.[34]

## COGNITIVE THEORY

Cognitive interpretation of SIB centers around frustration. It is suggested that blocking of needs that are ubiquitous in all human beings leads to frustration and may result in aggressive expression. If expression of aggression is blocked either by social authority or inadequate avenues, it gets turned inwards resulting in various compensatory behavior including self-injury.

In the first group, frustration may be coupled with helplessness, feeling of empowerment in situations of helplessness, venting of anger influencing others, establishing control, an act of cry for help.[72,63] In this group the threshold for tolerance is not altered and only when the threshold crossed there is frustration. Thus, there is temporary loss of impulse control and therefore they do not usually indulge in repetitive compensatory acts. It could also be a strategy of attention seeking and manipulative behavior[6] to satisfy one's wish through emotional black mail.[3] This behavior may also be employed to escape from unpleasant situations and duties.[7]

In the second group, the threshold to tolerate frustration may be reduced and minor and insignificant events evoke intolerable frustration. These patients being highly vulnerable to frustration to minor events are prone to repetitive SIB. In cases of self-injury, frustration, aggression and impulsivity may have different breakpoints in individual cases based on psychopathology specific to individual cases. A “self-harm personality profile” has been delineated with characteristics of strong and intense emotion with a heightened sensitivity to interpersonal rejection.[2] Time-limited cognitive behavioral intervention is effective for patients with chronic and recurrent self-harm.[81]

## LEARNING THEORY

According to learning theory, SIB is a behavioral problem maintained as an escape behavior controlled by negative reinforcement processes or attention seeking behavior controlled by positive reinforcement processes.[82]

## TRANSACTIONAL VIEW OF SIB

Transactional view of SIB suggests that there is interaction between physical, biochemical, social and interpersonal environments. According to this theory, SIB occurs under specific conditions of instigation that are unique to each person. SIB is a learned behavior representing a coping response that is reinforced by the effects produced by self-injurious acts. Risk factors for SIB include personal characteristics, genetic disorders, psychiatric disorders and general medical conditions. This integrative model encompassing instigative, central processing influences and maintaining conditions advocate comprehensive approach in the management of SIB.

**Figure 1:**
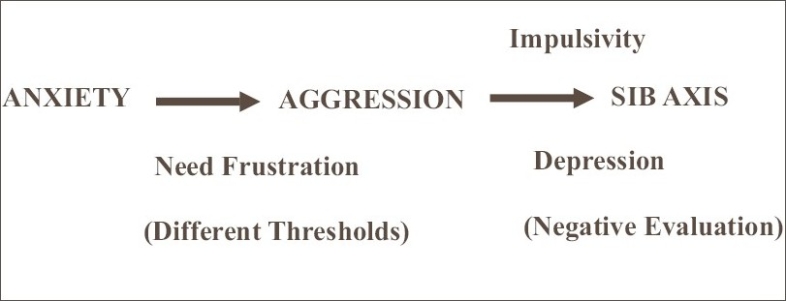
Comprehensive model of explanation based on psychopathology

**Figure 2:**
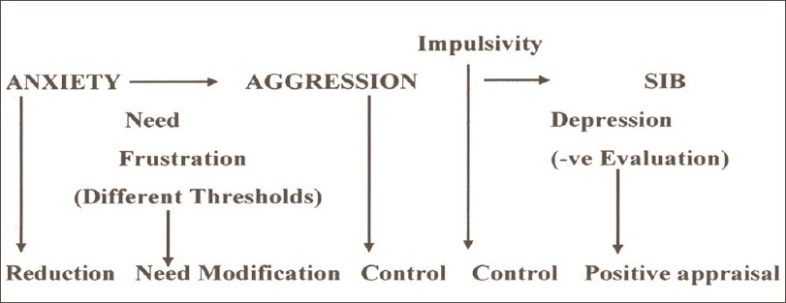
Treatment strategies based on psychopathology

Based on psychopathological considerations, a comprehensive model of explanation can be worked out as depicted in [Figure 1]. This model helps for planning treatment strategies, as suggested in [Figure 2].

## BIOCHEMICAL ASPECTS

Neurochemical explanations for SIB have been postulated to involve dopamine, opiate, serotonin and diazepam binding site. Some studies with markers have associated aggression and impulsiveness particularly associated with violent behavior with the altered serotonergic function.[83,84] Decreased serotonergic activity has been demonstrated in cases of borderline personality disorder, major depressive disorder, obsessive compulsive neurosis, and mental retardation who have concomitant impulsiveness, aggression and SIB.[85] Investigators have suggested opiate release, especially in repetitive self-mutilators.

In mental retardation, where the SIB is compulsive and stereotyped as in syndromes such as Lesch-Nyhan syndrome and Cornelia de Lange syndrome, neurochemical explanation of self-injurious behavior have been focused on dopaminergic dysregulation and dopamine receptor super sensitivity[86–90] as also in Tourette syndrome.[91,92] Both dopaminergic and serotonergic systems have been implicated in patients of mental retardation with psychosis.[93]

## CONCLUSION

In this paper, common types of SIB seen in an Indian General hospital set up have been reported. It is suggested that definition of SIB should be restricted to phenomenological explanation and the exclusion of cases of suicidal intention untenable.

In line with opinion of Roger and Pullen, and Ashok Singh, it is agreed that self-injurious behavior is not a single clinical entity and occurs in various psychiatric syndromes.[38,35] Clinically, most cases of self-injurious behavior will be part of some psychiatric diagnosis and the psychopathology will be of the respective psychiatric syndromes.

Cases of SIB have been categorized into three main clinical categories. The first group consists of isolated and mild form of SIB occurring mostly in normal or subthreshold psychiatric conditions. The second group consists of repetitive and moderately severe form of SIB occurring mainly in personality disorders and other nonpsychotic conditions. The third group consists of isolated and very severe form of SIB occurring in cases of psychoses and severe psychopathology

SIB seems to have mythological, religious, historical and cultural undertones, especially in normal and sub threshold psychiatric conditions. Psychopathological explanations of clinically relevant cases of SIB include psychoanalytical, cognitive and neurochemical models. Frustration, aggression, and impulsivity appearing in helpless situations appear to be a common script across all these models. The inherent needs, sexual or aggressive, may not have avenues for safe expression. This situation and blocking of needs desired in social situations and or in fantasy may trigger various forms of SIB based on these psychological states.

The severity of injury is determined by the extent of psychopathology. Site of injury and body part on which the injury is caused may have symbolic significance for a particular patient. Understanding of these clinicopsychopathological issues of SIB is of great help in management of such cases.
